# 8,9-Dimeth­oxy-5-phenyl­sulfonyl-5*H*-benzo[*b*]carbazole

**DOI:** 10.1107/S160053681001247X

**Published:** 2010-04-14

**Authors:** T. Kavitha, M. Thenmozhi, V. Dhayalan, A. K. Mohanakrishnan, M. N. Ponnuswamy

**Affiliations:** a Centre of Advanced Study in Crystallography and Biophysics, University of Madras, Guindy Campus, Chennai 600 025, India; b Department of Organic Chemistry, University of Madras, Guindy Campus, Chennai 600 025, India

## Abstract

In the title compound, C_24_H_19_NO_4_S, the benzocarbazole ring system is planar (r.m.s. deviation = 0.016 Å) and forms a dihedral angle of 78.54 (4)° with the sulfonyl-bound phenyl ring. Intra­molecular C—H⋯O inter­actions are observed. A *C*(8) chain running along the *b* axis is formed *via* inter­molecular C—H⋯O hydrogen bonds. The chains are linked *via* weak C—H⋯ π inter­actions.

## Related literature

For bond-length data, see: Allen *et al.* (1987 ▶). For the biological activity of carbazole derivatives, see: Itoigawa *et al.* (2000 ▶); Tachibana *et al.* (2001 ▶); Ramsewak *et al.* (1999 ▶). For related structures, see: Chakkaravarthi *et al.* (2008 ▶); Govindasamy *et al.* (1998 ▶).
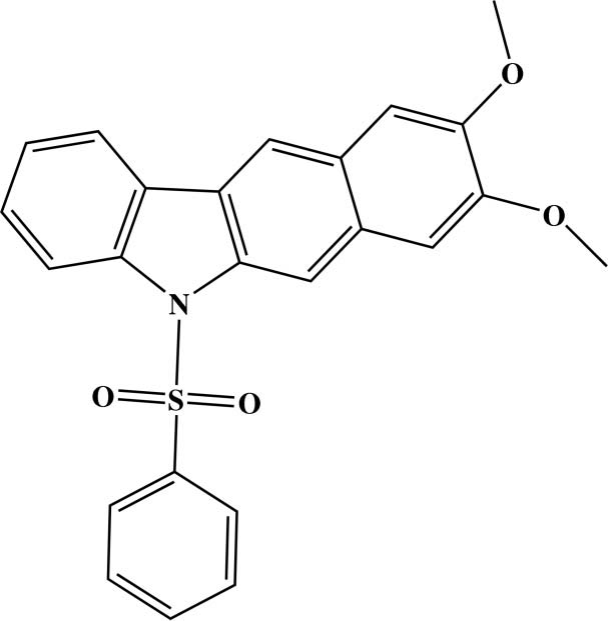


## Experimental

### 

#### Crystal data

C_24_H_19_NO_4_S
*M* *_r_* = 417.46
Triclinic, 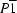

*a* = 7.8606 (2) Å
*b* = 9.5892 (2) Å
*c* = 13.8846 (4) Å
α = 100.387 (1)°
β = 93.168 (2)°
γ = 105.883 (1)°
*V* = 984.05 (4) Å^3^
*Z* = 2
Mo *K*α radiation
μ = 0.20 mm^−1^
*T* = 293 K
0.30 × 0.25 × 0.20 mm

#### Data collection

Bruker Kappa APEXII area-detector diffractometer
Absorption correction: multi-scan (*SADABS*; Sheldrick, 2001 ▶) *T*_min_ = 0.943, *T*_max_ = 0.962
26246 measured reflections
7025 independent reflections
5372 reflections with *I* > 2σ(*I*)
*R*_int_ = 0.024

#### Refinement

*R*[*F* ^2^ > 2σ(*F* ^2^)] = 0.044
*wR*(*F* ^2^) = 0.138
*S* = 1.04
7025 reflections
273 parameters
H-atom parameters constrained
Δρ_max_ = 0.39 e Å^−3^
Δρ_min_ = −0.25 e Å^−3^

### 

Data collection: *APEX2* (Bruker, 2004 ▶); cell refinement: *SAINT* (Bruker, 2004 ▶); data reduction: *SAINT*; program(s) used to solve structure: *SHELXS97* (Sheldrick, 2008 ▶); program(s) used to refine structure: *SHELXL97* (Sheldrick, 2008 ▶); molecular graphics: *ORTEP-3* (Farrugia, 1997 ▶); software used to prepare material for publication: *SHELXL97* and *PLATON* (Spek, 2009 ▶).

## Supplementary Material

Crystal structure: contains datablocks I, global. DOI: 10.1107/S160053681001247X/ci5059sup1.cif

Structure factors: contains datablocks I. DOI: 10.1107/S160053681001247X/ci5059Isup2.hkl

Additional supplementary materials:  crystallographic information; 3D view; checkCIF report

## Figures and Tables

**Table 1:** Hydrogen-bond geometry (Å, °)

*D*—H⋯*A*	*D*—H	H⋯*A*	*D*⋯*A*	*D*—H⋯*A*
C4—H4⋯O1	0.93	2.34	2.9238 (19)	120
C6—H6⋯O2	0.93	2.37	2.9674 (15)	122
C2—H2⋯O2^i^	0.93	2.55	3.3361 (17)	143
C24—H24*C*⋯*Cg*1^ii^	0.96	2.84	3.727 (2)	154
C25—H25*A*⋯*Cg*2^iii^	0.96	2.90	3.628 (2)	134

Symmetry codes: (i) 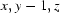; (ii) 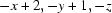; (iii) 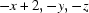. *Cg*1 and *Cg*2 are the centroids of the C7–C10/C22/C23 and C1–C4/C21/C20 rings, respectively.

**Fig. 1.**
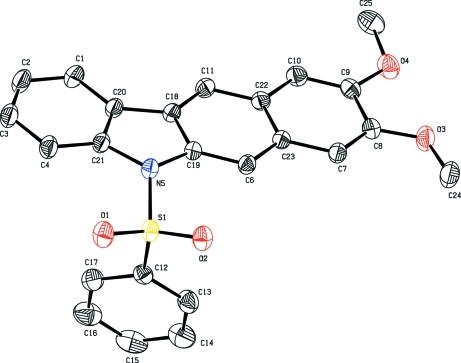
The molecular structure of the title compound. Displacement ellipsoids are drawn at the 30% probability level. H atoms have been omitted for clarity.

**Fig. 2.**
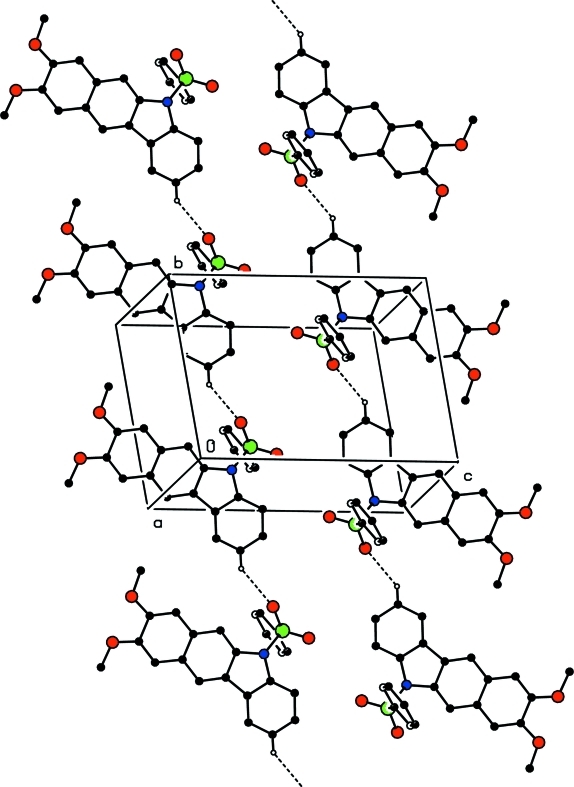
Crystal packing of the title compound. H atoms not involved in hydrogen bonding (dashed lines) have been omitted for clarity.

## supplementary crystallographic information

### Comment

Carbazole and its derivatives are considered as potential compounds owing to their applications in pharmacy and molecular electronics. They also possess various biological activities such as antitumor (Itoigawa *et al.*, 2000), antioxidative (Tachibana *et al.*, 2001), anti-inflammatory and antimutagenic (Ramsewak *et al.*, 1999) properties.

The benzocarbazole ring system is planar (r.m.s. deviation 0.016 Å) and it forms a dihedral angle of 78.54 (4)° with the sulfonyl-bound phenyl ring (Fig.1). The N—C bond lengths, namely N5—C19 and N5—C21 [1.432 (1) & 1.436 (2) Å] deviate slightly from the mean value reported in the literature (1.370 (12) Å; Allen *et al.*, 1987). This may be due to the electron-withdrawing character of the phenylsulfonyl group (Govindasamy *et al.*, 1998) substitued at N atom of the carbazole group. The S atom exhibits a distorted tetrahedral geometry. The widening of the O1—S1—O2 [119.60 (6)°] angle may be due to repulsive interactions between the two S═O bonds (Chakkaravarthi *et al.*, 2008). The sum of the bond angles around N1 [350.6°] indicate the sp^2^ hybridization. The methoxy groups substituted at C8 and C9 lie almost in the plane of the attached benzene ring [C7—C8—O3—C24 = -3.8 (2)° and C10—C9—O4—C25 = 6.8 (2)°].

The intermolecular C—H···O hydrogen bonds link the molecules into a C(8) chain running along the *b* axis (Fig.2). The packing of the molecules is further influenced by C—H··· π interactions.

### Experimental

To a solution of diethyl-2-[(2-bromomethyl-1-phenylsulfonyl-1*H*-indol-3-yl)methylene]malonate (0.41 g, 0.78 mmol) in dry 1,2-dichloroethane (15 ml), anhydrous ZnBr_2_ (0.35 g, 1.55 mmol) and veratrole (0.12 ml, 0.94 mmol) were added. The mixture was refluxed for 5 h under N_2_ atmosphere. The solvent was removed and the reaction mixture was quenched with ice-water (50 ml) containing 1 ml of conc. HCl, extracted with chloroform (2 × 10 ml) and dried (Na_2_SO_4_). Removal of the solvent followed by flash column chromatographic purification (n-hexane-ethyl acetate 99:1) led to the isolation of the title compound as a colourless solid. The compound was recrystallized from CDCl_3_.

### Refinement

H atoms were positioned geometrically (C–H = 0.93–0.96 Å) and allowed to ride on their parent atoms, with *U*_iso_(H) = 1.5*U*_eq_(C_methyl_) and 1.2*U*_eq_(C). A rotating group model was used for the methyl groups.

### Crystal data

**Table:**

C_24_H_19_NO_4_S	*Z* = 2
*M**_r_* = 417.46	*F*(000) = 436
Triclinic, *P*1	*D*_x_ = 1.409 Mg m^−^^3^
Hall symbol: -P 1	Mo *K*α radiation, λ = 0.71073 Å
*a* = 7.8606 (2) Å	Cell parameters from 7025 reflections
*b* = 9.5892 (2) Å	θ = 1.5–33.3°
*c* = 13.8846 (4) Å	µ = 0.20 mm^−^^1^
α = 100.387 (1)°	*T* = 293 K
β = 93.168 (2)°	Block, colourless
γ = 105.883 (1)°	0.30 × 0.25 × 0.20 mm
*V* = 984.05 (4) Å^3^	

### Data collection

**Table:**

Bruker Kappa APEXII area-detector diffractometer	7025 independent reflections
Radiation source: fine-focus sealed tube	5372 reflections with *I* > 2σ(*I*)
graphite	*R*_int_ = 0.024
ω and φ scans	θ_max_ = 33.3°, θ_min_ = 1.5°
Absorption correction: multi-scan (*SADABS*; Sheldrick, 2001)	*h* = −12→11
*T*_min_ = 0.943, *T*_max_ = 0.962	*k* = −14→13
26246 measured reflections	*l* = −21→21

### Refinement

**Table:**

Refinement on *F*^2^	Primary atom site location: structure-invariant direct methods
Least-squares matrix: full	Secondary atom site location: difference Fourier map
*R*[*F*^2^ > 2σ(*F*^2^)] = 0.044	Hydrogen site location: inferred from neighbouring sites
*wR*(*F*^2^) = 0.138	H-atom parameters constrained
*S* = 1.04	*w* = 1/[σ^2^(*F*_o_^2^) + (0.0749*P*)^2^ + 0.1333*P*] where *P* = (*F*_o_^2^ + 2*F*_c_^2^)/3
7025 reflections	(Δ/σ)_max_ = 0.001
273 parameters	Δρ_max_ = 0.39 e Å^−^^3^
0 restraints	Δρ_min_ = −0.25 e Å^−^^3^

### Special details

**Table:**

Geometry. All esds (except the esd in the dihedral angle between two l.s. planes) are estimated using the full covariance matrix. The cell esds are taken into account individually in the estimation of esds in distances, angles and torsion angles; correlations between esds in cell parameters are only used when they are defined by crystal symmetry. An approximate (isotropic) treatment of cell esds is used for estimating esds involving l.s. planes.
Refinement. Refinement of *F*^2^ against ALL reflections. The weighted *R*-factor wR and goodness of fit *S* are based on *F*^2^, conventional *R*-factors *R* are based on *F*, with *F* set to zero for negative *F*^2^. The threshold expression of *F*^2^ > σ(*F*^2^) is used only for calculating *R*-factors(gt) etc. and is not relevant to the choice of reflections for refinement. *R*-factors based on *F*^2^ are statistically about twice as large as those based on *F*, and *R*- factors based on ALL data will be even larger.

### Fractional atomic coordinates and isotropic or equivalent isotropic displacement parameters (Å^2^)

**Table:**

	*x*	*y*	*z*	*U*_iso_*/*U*_eq_	
S1	0.62768 (4)	0.24308 (3)	0.35159 (2)	0.03860 (9)	
O1	0.54424 (14)	0.18104 (12)	0.42890 (8)	0.0543 (3)	
O2	0.57390 (13)	0.35798 (11)	0.31820 (7)	0.0483 (2)	
O3	0.84578 (17)	0.47850 (12)	−0.16011 (8)	0.0580 (3)	
O4	0.89925 (15)	0.24214 (13)	−0.25315 (7)	0.0566 (3)	
C1	0.6666 (2)	−0.24922 (15)	0.16579 (12)	0.0538 (3)	
H1	0.7018	−0.2941	0.1083	0.065*	
C2	0.6256 (2)	−0.32426 (16)	0.24138 (14)	0.0600 (4)	
H2	0.6350	−0.4200	0.2351	0.072*	
C3	0.5710 (2)	−0.25904 (16)	0.32592 (13)	0.0543 (4)	
H3	0.5440	−0.3121	0.3757	0.065*	
C4	0.55495 (18)	−0.11624 (15)	0.33915 (11)	0.0484 (3)	
H4	0.5166	−0.0732	0.3962	0.058*	
N5	0.59104 (14)	0.10465 (11)	0.25487 (8)	0.0387 (2)	
C6	0.67353 (17)	0.25195 (13)	0.12110 (9)	0.0386 (2)	
H6	0.6486	0.3362	0.1542	0.046*	
C7	0.76027 (18)	0.37119 (14)	−0.01873 (9)	0.0421 (3)	
H7	0.7390	0.4572	0.0142	0.051*	
C8	0.81631 (18)	0.36643 (15)	−0.11016 (9)	0.0430 (3)	
C9	0.84830 (17)	0.23465 (15)	−0.16184 (9)	0.0432 (3)	
C10	0.82608 (18)	0.11485 (15)	−0.11883 (9)	0.0442 (3)	
H10	0.8490	0.0300	−0.1525	0.053*	
C11	0.74574 (18)	−0.00587 (13)	0.02134 (10)	0.0424 (3)	
H11	0.7687	−0.0913	−0.0112	0.051*	
C12	0.85883 (16)	0.30130 (14)	0.38223 (8)	0.0386 (2)	
C13	0.9589 (2)	0.42875 (18)	0.35613 (11)	0.0530 (3)	
H13	0.9045	0.4838	0.3228	0.064*	
C14	1.1410 (2)	0.4730 (2)	0.38032 (12)	0.0677 (5)	
H14	1.2102	0.5592	0.3641	0.081*	
C15	1.2202 (2)	0.3895 (2)	0.42836 (13)	0.0687 (5)	
H15	1.3429	0.4197	0.4445	0.082*	
C16	1.1192 (3)	0.2617 (2)	0.45275 (15)	0.0726 (5)	
H16	1.1743	0.2055	0.4845	0.087*	
C17	0.9365 (2)	0.21628 (18)	0.43036 (12)	0.0575 (4)	
H17	0.8674	0.1306	0.4473	0.069*	
C18	0.68934 (16)	0.00057 (12)	0.11331 (9)	0.0379 (2)	
C19	0.65257 (15)	0.12981 (12)	0.16256 (8)	0.0348 (2)	
C20	0.65429 (16)	−0.10571 (13)	0.17721 (10)	0.0406 (2)	
C21	0.59885 (15)	−0.04054 (13)	0.26328 (10)	0.0396 (2)	
C22	0.76869 (16)	0.11715 (13)	−0.02368 (9)	0.0382 (2)	
C23	0.73374 (16)	0.24697 (12)	0.02715 (8)	0.0368 (2)	
C24	0.8057 (3)	0.60947 (18)	−0.11663 (13)	0.0630 (4)	
H24A	0.6826	0.5860	−0.1055	0.094*	
H24B	0.8287	0.6784	−0.1600	0.094*	
H24C	0.8788	0.6528	−0.0550	0.094*	
C25	0.9127 (2)	0.1091 (2)	−0.31191 (12)	0.0639 (4)	
H25A	1.0028	0.0769	−0.2805	0.096*	
H25B	0.9438	0.1264	−0.3755	0.096*	
H25C	0.8005	0.0341	−0.3195	0.096*	

### Atomic displacement parameters (Å^2^)

**Table:**

	*U*^11^	*U*^22^	*U*^33^	*U*^12^	*U*^13^	*U*^23^
S1	0.04190 (16)	0.04201 (16)	0.03897 (15)	0.01777 (12)	0.01525 (11)	0.01425 (11)
O1	0.0600 (6)	0.0618 (6)	0.0504 (5)	0.0199 (5)	0.0287 (5)	0.0245 (5)
O2	0.0553 (5)	0.0502 (5)	0.0523 (5)	0.0299 (4)	0.0170 (4)	0.0168 (4)
O3	0.0840 (8)	0.0512 (6)	0.0463 (5)	0.0219 (5)	0.0177 (5)	0.0221 (4)
O4	0.0694 (7)	0.0673 (7)	0.0392 (5)	0.0250 (5)	0.0174 (5)	0.0151 (4)
C1	0.0658 (9)	0.0351 (6)	0.0620 (9)	0.0165 (6)	0.0024 (7)	0.0124 (6)
C2	0.0685 (9)	0.0359 (6)	0.0768 (11)	0.0123 (6)	−0.0037 (8)	0.0224 (7)
C3	0.0503 (7)	0.0453 (7)	0.0676 (9)	0.0041 (6)	−0.0019 (6)	0.0296 (7)
C4	0.0456 (6)	0.0460 (7)	0.0551 (7)	0.0068 (5)	0.0065 (6)	0.0241 (6)
N5	0.0430 (5)	0.0359 (5)	0.0405 (5)	0.0118 (4)	0.0081 (4)	0.0140 (4)
C6	0.0494 (6)	0.0326 (5)	0.0367 (5)	0.0152 (4)	0.0084 (5)	0.0084 (4)
C7	0.0546 (7)	0.0358 (5)	0.0382 (6)	0.0146 (5)	0.0078 (5)	0.0103 (4)
C8	0.0482 (6)	0.0444 (6)	0.0377 (6)	0.0121 (5)	0.0051 (5)	0.0134 (5)
C9	0.0442 (6)	0.0520 (7)	0.0340 (5)	0.0147 (5)	0.0057 (5)	0.0088 (5)
C10	0.0513 (7)	0.0448 (6)	0.0381 (6)	0.0186 (5)	0.0072 (5)	0.0044 (5)
C11	0.0524 (7)	0.0340 (5)	0.0428 (6)	0.0173 (5)	0.0050 (5)	0.0054 (4)
C12	0.0426 (6)	0.0437 (6)	0.0313 (5)	0.0159 (5)	0.0085 (4)	0.0052 (4)
C13	0.0522 (7)	0.0612 (8)	0.0453 (7)	0.0092 (6)	0.0095 (6)	0.0198 (6)
C14	0.0506 (8)	0.0878 (12)	0.0531 (8)	−0.0009 (8)	0.0129 (7)	0.0152 (8)
C15	0.0437 (7)	0.0943 (13)	0.0579 (9)	0.0186 (8)	0.0040 (7)	−0.0083 (9)
C16	0.0676 (10)	0.0737 (11)	0.0762 (12)	0.0361 (9)	−0.0170 (9)	−0.0025 (9)
C17	0.0611 (8)	0.0498 (8)	0.0610 (9)	0.0184 (6)	−0.0082 (7)	0.0106 (6)
C18	0.0411 (6)	0.0313 (5)	0.0414 (6)	0.0105 (4)	0.0015 (4)	0.0084 (4)
C19	0.0370 (5)	0.0329 (5)	0.0356 (5)	0.0105 (4)	0.0036 (4)	0.0089 (4)
C20	0.0416 (6)	0.0327 (5)	0.0477 (6)	0.0089 (4)	−0.0004 (5)	0.0127 (5)
C21	0.0359 (5)	0.0349 (5)	0.0482 (6)	0.0065 (4)	0.0014 (5)	0.0157 (5)
C22	0.0417 (6)	0.0369 (5)	0.0362 (5)	0.0129 (4)	0.0036 (4)	0.0060 (4)
C23	0.0425 (6)	0.0337 (5)	0.0344 (5)	0.0112 (4)	0.0042 (4)	0.0074 (4)
C24	0.0872 (12)	0.0493 (8)	0.0610 (9)	0.0243 (8)	0.0164 (8)	0.0236 (7)
C25	0.0695 (10)	0.0806 (11)	0.0418 (7)	0.0277 (8)	0.0139 (7)	0.0017 (7)

### Geometric parameters (Å, °)

**Table:**

S1—O2	1.4231 (10)	C9—C10	1.3623 (19)
S1—O2	1.4231 (10)	C10—C22	1.4177 (17)
S1—O1	1.4251 (9)	C10—H10	0.93
S1—O1	1.4251 (9)	C11—C18	1.3712 (17)
S1—N5	1.6594 (11)	C11—C22	1.4060 (17)
S1—C12	1.7515 (13)	C11—H11	0.93
O3—C8	1.3564 (15)	C12—C17	1.3811 (19)
O3—C24	1.416 (2)	C12—C13	1.3816 (19)
O4—C9	1.3587 (15)	C13—C14	1.380 (2)
O4—C25	1.4183 (19)	C13—H13	0.93
C1—C2	1.381 (2)	C14—C15	1.376 (3)
C1—C20	1.3872 (18)	C14—H14	0.93
C1—H1	0.93	C15—C16	1.376 (3)
C2—C3	1.375 (3)	C15—H15	0.93
C2—H2	0.93	C16—C17	1.381 (2)
C3—C4	1.390 (2)	C16—H16	0.93
C3—H3	0.93	C17—H17	0.93
C4—C21	1.3909 (17)	C18—C19	1.4139 (16)
C4—H4	0.93	C18—C20	1.4494 (16)
N5—C19	1.4321 (14)	C20—C21	1.3950 (19)
N5—C21	1.4355 (15)	C22—C23	1.4196 (17)
C6—C19	1.3705 (15)	C24—H24A	0.96
C6—C23	1.4093 (16)	C24—H24B	0.96
C6—H6	0.93	C24—H24C	0.96
C7—C8	1.3637 (17)	C25—H25A	0.96
C7—C23	1.4215 (16)	C25—H25B	0.96
C7—H7	0.93	C25—H25C	0.96
C8—C9	1.4289 (19)		
			
O2—S1—O1	119.60 (6)	C14—C13—C12	118.85 (16)
O2—S1—N5	106.42 (6)	C14—C13—H13	120.6
O1—S1—N5	106.38 (6)	C12—C13—H13	120.6
O2—S1—C12	109.08 (6)	C15—C14—C13	120.03 (17)
O2—S1—C12	109.08 (6)	C15—C14—H14	120.0
O1—S1—C12	109.33 (6)	C13—C14—H14	120.0
O1—S1—C12	109.33 (6)	C14—C15—C16	120.53 (15)
N5—S1—C12	105.02 (5)	C14—C15—H15	119.7
C8—O3—C24	117.66 (12)	C16—C15—H15	119.7
C9—O4—C25	116.75 (12)	C15—C16—C17	120.44 (17)
C2—C1—C20	118.78 (15)	C15—C16—H16	119.8
C2—C1—H1	120.6	C17—C16—H16	119.8
C20—C1—H1	120.6	C12—C17—C16	118.38 (16)
C3—C2—C1	120.75 (14)	C12—C17—H17	120.8
C3—C2—H2	119.6	C16—C17—H17	120.8
C1—C2—H2	119.6	C11—C18—C19	120.27 (11)
C2—C3—C4	121.93 (14)	C11—C18—C20	132.36 (11)
C2—C3—H3	119.0	C19—C18—C20	107.37 (11)
C4—C3—H3	119.0	C6—C19—C18	121.62 (11)
C3—C4—C21	117.01 (15)	C6—C19—N5	129.82 (10)
C3—C4—H4	121.5	C18—C19—N5	108.55 (9)
C21—C4—H4	121.5	C1—C20—C21	120.01 (12)
C19—N5—C21	107.00 (9)	C1—C20—C18	131.88 (13)
C19—N5—S1	120.99 (8)	C21—C20—C18	108.10 (11)
C21—N5—S1	122.60 (8)	C4—C21—C20	121.51 (12)
C19—C6—C23	118.47 (11)	C4—C21—N5	129.56 (13)
C19—C6—H6	120.8	C20—C21—N5	108.88 (10)
C23—C6—H6	120.8	C11—C22—C10	121.58 (11)
C8—C7—C23	121.15 (12)	C11—C22—C23	119.36 (11)
C8—C7—H7	119.4	C10—C22—C23	119.06 (11)
C23—C7—H7	119.4	C6—C23—C22	120.47 (11)
O3—C8—C7	125.72 (13)	C6—C23—C7	120.86 (11)
O3—C8—C9	114.26 (11)	C22—C23—C7	118.67 (11)
C7—C8—C9	120.02 (12)	O3—C24—H24A	109.5
O4—C9—C10	125.54 (12)	O3—C24—H24B	109.5
O4—C9—C8	114.56 (12)	H24A—C24—H24B	109.5
C10—C9—C8	119.89 (12)	O3—C24—H24C	109.5
C9—C10—C22	121.20 (12)	H24A—C24—H24C	109.5
C9—C10—H10	119.4	H24B—C24—H24C	109.5
C22—C10—H10	119.4	O4—C25—H25A	109.5
C18—C11—C22	119.80 (11)	O4—C25—H25B	109.5
C18—C11—H11	120.1	H25A—C25—H25B	109.5
C22—C11—H11	120.1	O4—C25—H25C	109.5
C17—C12—C13	121.76 (13)	H25A—C25—H25C	109.5
C17—C12—S1	118.76 (11)	H25B—C25—H25C	109.5
C13—C12—S1	119.47 (11)		
			
O2—S1—O1—O1	0.00 (16)	C13—C14—C15—C16	0.0 (3)
O2—S1—O1—O1	0.00 (16)	C14—C15—C16—C17	−0.8 (3)
N5—S1—O1—O1	0.00 (14)	C13—C12—C17—C16	0.2 (2)
C12—S1—O1—O1	0.00 (17)	S1—C12—C17—C16	178.91 (12)
O1—S1—O2—O2	0.00 (14)	C15—C16—C17—C12	0.7 (3)
O1—S1—O2—O2	0.00 (14)	C22—C11—C18—C19	0.61 (19)
N5—S1—O2—O2	0.00 (15)	C22—C11—C18—C20	−179.99 (12)
C12—S1—O2—O2	0.00 (16)	C23—C6—C19—C18	−0.16 (18)
C20—C1—C2—C3	0.9 (2)	C23—C6—C19—N5	−178.68 (11)
C1—C2—C3—C4	−0.1 (2)	C11—C18—C19—C6	−0.69 (18)
C2—C3—C4—C21	−0.8 (2)	C20—C18—C19—C6	179.78 (11)
O2—S1—N5—C19	49.36 (10)	C11—C18—C19—N5	178.11 (11)
O2—S1—N5—C19	49.36 (10)	C20—C18—C19—N5	−1.42 (13)
O1—S1—N5—C19	177.92 (9)	C21—N5—C19—C6	−178.66 (12)
O1—S1—N5—C19	177.92 (9)	S1—N5—C19—C6	−31.42 (17)
C12—S1—N5—C19	−66.23 (10)	C21—N5—C19—C18	2.67 (12)
O2—S1—N5—C21	−168.55 (9)	S1—N5—C19—C18	149.90 (9)
O2—S1—N5—C21	−168.55 (9)	C2—C1—C20—C21	−0.9 (2)
O1—S1—N5—C21	−39.99 (11)	C2—C1—C20—C18	179.24 (14)
O1—S1—N5—C21	−39.99 (11)	C11—C18—C20—C1	0.0 (2)
C12—S1—N5—C21	75.86 (10)	C19—C18—C20—C1	179.45 (14)
C24—O3—C8—C7	−3.8 (2)	C11—C18—C20—C21	−179.87 (13)
C24—O3—C8—C9	175.91 (13)	C19—C18—C20—C21	−0.41 (13)
C23—C7—C8—O3	−179.89 (12)	C3—C4—C21—C20	0.81 (19)
C23—C7—C8—C9	0.4 (2)	C3—C4—C21—N5	178.13 (12)
C25—O4—C9—C10	6.8 (2)	C1—C20—C21—C4	0.02 (19)
C25—O4—C9—C8	−172.91 (13)	C18—C20—C21—C4	179.91 (11)
O3—C8—C9—O4	−1.32 (17)	C1—C20—C21—N5	−177.80 (12)
C7—C8—C9—O4	178.41 (12)	C18—C20—C21—N5	2.09 (13)
O3—C8—C9—C10	179.00 (12)	C19—N5—C21—C4	179.47 (12)
C7—C8—C9—C10	−1.3 (2)	S1—N5—C21—C4	32.88 (17)
O4—C9—C10—C22	−178.64 (12)	C19—N5—C21—C20	−2.94 (13)
C8—C9—C10—C22	1.0 (2)	S1—N5—C21—C20	−149.53 (9)
O2—S1—C12—C17	170.30 (11)	C18—C11—C22—C10	−179.74 (11)
O2—S1—C12—C17	170.30 (11)	C18—C11—C22—C23	0.28 (19)
O1—S1—C12—C17	37.83 (12)	C9—C10—C22—C11	−179.87 (12)
O1—S1—C12—C17	37.83 (12)	C9—C10—C22—C23	0.11 (19)
N5—S1—C12—C17	−75.97 (12)	C19—C6—C23—C22	1.07 (18)
O2—S1—C12—C13	−10.97 (13)	C19—C6—C23—C7	−179.10 (12)
O2—S1—C12—C13	−10.97 (13)	C11—C22—C23—C6	−1.14 (18)
O1—S1—C12—C13	−143.44 (11)	C10—C22—C23—C6	178.88 (11)
O1—S1—C12—C13	−143.44 (11)	C11—C22—C23—C7	179.02 (12)
N5—S1—C12—C13	102.76 (11)	C10—C22—C23—C7	−0.95 (18)
C17—C12—C13—C14	−1.0 (2)	C8—C7—C23—C6	−179.15 (12)
S1—C12—C13—C14	−179.69 (12)	C8—C7—C23—C22	0.7 (2)
C12—C13—C14—C15	0.9 (2)		

### Hydrogen-bond geometry (Å, °)

**Table:**

*D*—H···*A*	*D*—H	H···*A*	*D*···*A*	*D*—H···*A*
C4—H4···O1	0.93	2.34	2.9238 (19)	120
C6—H6···O2	0.93	2.37	2.9674 (15)	122
C2—H2···O2^i^	0.93	2.55	3.3361 (17)	143
C24—H24C···Cg1^ii^	0.96	2.84	3.7272 (21)	154
C25—H25A···Cg2^iii^	0.96	2.90	3.6276 (21)	134

Symmetry codes:  (i) *x*, *y*−1, *z*; (ii) −*x*+2, −*y*+1, −*z*; (iii) −*x*+2, −*y*, −*z*.
